# Libraries of the World

**Published:** 1887-09

**Authors:** 


### LIBRARIES OF THE WORLD.

The largest library in the world is that of the French, at Paris, which contains to-day upward of 2,000,000 printed books and 160,000 manuscripts. Between the Imperial Library at St. Petersburg and the British Museum, it is difficult to say which is the larger. Neither will vary much from 1,100,000 volumes. The Royal Library of Munich has now something over 900,000, but this includes 500,000 pamphlets ; the Royal Library at Berlin contains 700,000 ; the library at Copenhagen, 510,000; the library at Dresden, 500,000 ; the library at Vienna, 400,000 ; the University Library at Gottingen, Germany, 400,000. The Vatican Library at Rome has about 120,000 printed books, and was commenced in 1378.

There are about sixty other libraries in Europe larger than the Vatican Library. The National Library of Paris is one of the very oldest in Europe' having been founded in 1350, although the University Library of Prague is reported founded the same year. The British Museum dates its commencement about four hundred years later—1763. Of the large libraries in the United States, the Boston Public Library comes next to the Congressional, with about 350,000 (including the duplicates in its seven branches) ; the Harvard University collection comes next, with about 210,000.

The Astor and Mercantile, of New York, have each about 150,000 ; Yale College has about 115,000 ; Dartmouth about 54,000 ; Cornell University has 42,000 ; the University of Virginia, 42,000 ; Bowdoin has 38,-

000 ; the University of South Carolina has 30,000 ; Michigan State, 40,000 ; Amherst, 44,500 ; Princeton, 45,000 ; Pennsylvania Mercantile, 126,000; and Columbia University, South Carolina, 32,000. It will thus be seen that our National Library, as it should be called, exceeds all but eight, or possibly nine, of the ancient libraries of Europe, and allin America.

